# Supplementary material for: Analysis of PPARγ Signaling Activity in Psoriasis
Source: Int J Mol Sci. 2021 Aug 10;22(16):8603. doi: 10.3390/ijms22168603 (PMC8395241; doi:10.3390/ijms22168603)
Supplement: Supplementary file 1 [file ijms-22-08603-s001.zip › Supplemental materials_Analysis of PPARg signaling activity in psoriasis/Pathway models/Models images and html files/PPARG inhibits phenotypes assotiated with psoriasis/143115.html]

cell stress


# Cell Process cell stress

|  |  |
| --- | --- |
| URN | urn:agi-cellproc:cell%20stress |
| Total Entities | 44 |
| Connectivity | 3914 |
| Name | cell stress |

---

|  |  |
| --- | --- |
| ChildConcepts | cellular stress response |
|  | oxidative stress |
|  | mitochondrial Ca2+ overload |
|  | cellular stress signal |
|  | electrophilic stress |
|  | endoplasmic reticulum stress |
|  | nitrative stress |
|  | nitrosative stress |
|  | redox stress |
|  | umbilical vein endothelial cell stress |
|  | endothelial cell stress |
|  | macrophage stress |
|  | erythroblast stress |
|  | burst forming unit E stress |
|  | kidney mesangial cell stress |
|  | lymphocyte stress |
|  | mast cell stress |
|  | immune system cell stress |
|  | hematopoietic cell stress |
|  | alveolar macrophage stress |
|  | monocyte stress |
|  | stem cell stress |
|  | microglia stress |
|  | myofibroblast stress |
|  | T-cell stress |
|  | neutrophil stress |
|  | blood platelet stress |
|  | plasmacytoid dendritic cell stress |
|  | lung fibroblast stress |
|  | reticulocyte stress |
|  | foam cell stress |
|  | leukocyte stress |
|  | B-cell stress |
|  | fibroblast stress |
|  | dendritic cell stress |
|  | granulocyte stress |
|  | M1 macrophage stress |
|  | PBMC stress |
|  | natural killer cell stress |
|  | erythroid precursor cell stress |
|  | phagocyte stress |
|  | mononuclear cell stress |
|  | cancer stem cell stress |
|  | immunocompetent cell stress |

---

|  |  |
| --- | --- |
| Pathway | PPARG inhibits phenotypes assotiated with psoriasis |

---

|  |  |
| --- | --- |
| MedScan ID | 4203677 |

---

|  |  |
| --- | --- |
| Alias | cellular stress |
|  | cellular stresses |
|  | cell stress |
|  | cell stresses |

---
